# High-Accuracy Decoupling Estimation of the Systematic Coordinate Errors of an INS and Intensified High Dynamic Star Tracker Based on the Constrained Least Squares Method

**DOI:** 10.3390/s17102285

**Published:** 2017-10-07

**Authors:** Jie Jiang, Wenbo Yu, Guangjun Zhang

**Affiliations:** Key Laboratory of Precision Opto-Mechatronics Technology, Ministry of Education, School of Instrumentation Science and Opto-Electronics Engineering, Beihang University, No. 37 Xueyuan Road, Haidian District, Beijing 100191, China; jiangjie@buaa.edu.cn (J.J.); guangjunzhang@buaa.edu.cn (G.Z.)

**Keywords:** inertial navigation system, attitude accuracy assessment, intensified high dynamic star tracker, installation error, misalignment error, decoupling estimation model, constrained least squares

## Abstract

Navigation accuracy is one of the key performance indicators of an inertial navigation system (INS). Requirements for an accuracy assessment of an INS in a real work environment are exceedingly urgent because of enormous differences between real work and laboratory test environments. An attitude accuracy assessment of an INS based on the intensified high dynamic star tracker (IHDST) is particularly suitable for a real complex dynamic environment. However, the coupled systematic coordinate errors of an INS and the IHDST severely decrease the attitude assessment accuracy of an INS. Given that, a high-accuracy decoupling estimation method of the above systematic coordinate errors based on the constrained least squares (CLS) method is proposed in this paper. The reference frame of the IHDST is firstly converted to be consistent with that of the INS because their reference frames are completely different. Thereafter, the decoupling estimation model of the systematic coordinate errors is established and the CLS-based optimization method is utilized to estimate errors accurately. After compensating for error, the attitude accuracy of an INS can be assessed based on IHDST accurately. Both simulated experiments and real flight experiments of aircraft are conducted, and the experimental results demonstrate that the proposed method is effective and shows excellent performance for the attitude accuracy assessment of an INS in a real work environment.

## 1. Introduction

An inertial navigation system (INS) can provide the position, velocity, and attitude knowledge of a carrier. It is an autonomous navigation system which does not rely on any external information or radiate energy to the outside, and thus it has been widely used in the military and civil fields [1,2,3,4,5]. Navigation accuracy is always one of the key performance indicators of an INS. In order to improve navigation accuracy, the error parameters of an INS should be accurately calibrated before its official use. The commonly used laboratory calibration methods are the multi-position method and its various improved methods [6,7,8]. However, when an INS works in a real dynamic environment, particularly for aircraft or other high maneuverability carriers, there always exist enormous differences between the real work and laboratory test environments. This will make the credibility of the accuracy assessment of an INS in the laboratory test environment decrease remarkably. Given that, the requirements for an accuracy assessment of an INS in a real complex dynamic environment are exceedingly urgent. So far, high-accuracy speed and position benchmarks can be provided by global positioning system (GPS) [9,10], and thus an accuracy assessment of the speed and position of an INS can be realized by comparing the output information of the INS with that of the GPS. However, a high-accuracy attitude benchmark in a real complex dynamic environment is still unavailable.

A star tracker is used to determine the attitude of a carrier by matching observation stars in the field of view (FOV) and guide stars in the star catalogue. A star tracker can reach an attitude accuracy on the arc-seconds level, and it also has the characteristic of being drift-free [11,12,13]. Given that, a star tracker can be used as a potential benchmark for an attitude accuracy assessment of an INS. However, the traditional star tracker is only suitable for approximate static conditions. Under dynamic conditions, a star spot continuously moves and forms a smeared star streak because of the long exposure time of the traditional star tracker. This will make the star spot energy disperse and the signal-to-noise ratio decrease, thus reducing the attitude accuracy and even failing to output the attitude information [14,15]. Fortunately, the dynamic performance of star trackers has been significantly improved in recent years with the development of photodetectors [16,17,18] and through progress in the dynamic performance-related algorithms of star trackers [19,20,21,22,23,24]. At present, the latest intensified high dynamic star tracker (IHDST) developed by the authors has reached an attitude accuracy on the arc-seconds level in a dynamic condition of up to 25°/s [25,26]. The IHDST can be used as the benchmark for an attitude accuracy assessment of an INS, which is particularly suitable for a real complex dynamic environment.

However, the systematic coordinate errors of an INS and IHDST, including the installation error between the INS and IHDST as well as the misalignment error of the INS, severely decrease the accuracy of the above assessment method, and the effects of the above two errors are coupled. In an integrated navigation system of an INS and a star tracker, the measurement models of the above two are known. Therefore, the existing method tends to utilize the above measurement models to establish the equations of the Karman filter or other similar filters and then estimate the systematic coordinate errors [27]. However, in this study, an attitude accuracy assessment of an INS based on the IHDST is the subject of concern. The INS becomes the assessed object, whose measurement model is unknown. At this time, the existing filter estimation methods are no longer applicable. Given that, a high-accuracy decoupling estimation method of the above systematic coordinate errors based on the constrained least squares (CLS) method is proposed in this paper. This method only utilizes the attitude data of an INS and IHDST to accurately estimate the above two errors without knowledge of the measurement models of the INS and IHDST. Since INSs and IHDSTs have completely different reference frames, the reference frame of the IHDST should be firstly converted to be consistent with that of the INS. Thereafter, the decoupling estimation model of the above two errors is established, and the CLS-based optimization method is then utilized to estimate them accurately. After compensating for the above two errors, the attitude accuracy of the INS can be ultimately assessed by using the IHDST as the attitude benchmark. Moreover, since the decoupling estimation model established and the CLS-based optimization method utilized can estimate the installation and misalignment errors accurately, the proposed method is also suitable for improving the accuracy of an INS and star tracker integrated navigation system [27,28,29].

The remainder of this paper is organized as follows. The unified principle of reference frames of the IHDST and an INS is deduced in Section 2. Section 3 details the decoupling estimation model and the CLS-based optimization method, which are utilized to estimate installation and misalignment errors accurately. Both simulated experiments and real flight experiments of an attitude accuracy assessment of an INS based on the IHDST are conducted in Section 4, and the experimental results demonstrate the feasibility and effectiveness of the proposed method. Finally, conclusions are drawn in Section 5.

## 2. Unified Principle of Reference Frame

The reference frame of the IHDST is entirely different from that of an INS due to their different measurement principles. Therefore, the reference frame of the IHDST should be converted to be consistent with that of an INS before the attitude accuracy assessment of the INS. The relevant coordinate frames are firstly defined for convenience, and then the conversion principle of a reference frame is deduced based on them.

### 2.1. Coordinate Frame Definition

**Figure 1:**
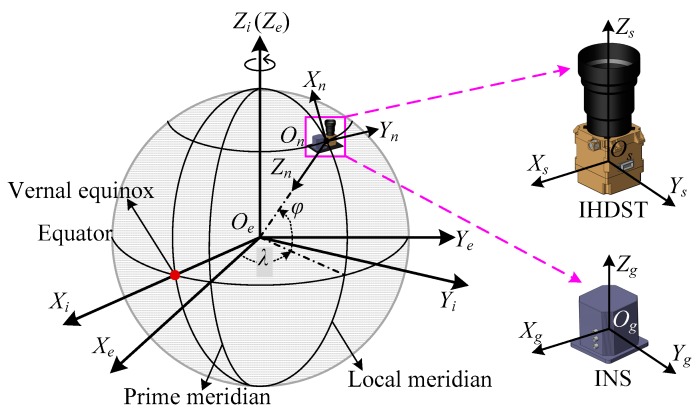
Relative position relations of the coordinate frames. INS: inertial navigation system. IHDST: intensified high dynamic star tracker.

The coordinate frames involved in this paper are shown in Figure 1, and they are defined as follows:

(1) The inertial frame *O_e_X_i_Y_i_Z_i_* (*i-frame*). The J2000.0 celestial coordinate frame, which is established at 12 terrestrial dynamical time on 1 January 2000, is selected as the inertial frame in this paper. The origin *O_e_* is set at the center of the earth; the *Z_i_*-axis is normal to the equatorial plane and points towards the north celestial pole; the *X_i_*-axis lies in the equatorial plane and points towards the vernal equinox at the establishment time; and the *Y_i_*-axis completes a right-handed orthogonal frame. The inertial frame is the reference frame of the IHDST.

(2) The earth fixed frame *O_e_X_e_Y_e_Z_e_* (*e-frame*). This frame is fixed with the earth and thus remains stationary relative to the earth. The origin *O_e_* is set at the center of the earth; the *Z_e_*-axis is normal to the equatorial plane and points towards the north celestial pole; the *X_e_*-axis lies in the equatorial plane and points towards the prime meridian at the observation time; and the *Y_e_*-axis completes a right-handed orthogonal frame.

(3) The navigation frame *O_n_X_n_Y_n_Z_n_* (*n-frame*). This frame is a local vertical frame and it is related to the local geographic latitude and longitude. The origin *O_n_* is set at the location of the carrier. The *X_n_*-axis points towards the north, the *Y_n_*-axis points towards the east, and the *Z_n_*-axis points downwards. The navigation frame is the reference frame of an INS.

(4) The IHDST frame *O_s_X_s_Y_s_Z_s_* (*s-frame*). The *X_s_*- and *Y_s_*-axes are parallel to the two vertical edges of the detector plane, respectively, the *Z_s_*-axis is along the boresight of the IHDST and points outwards, and the three axes satisfy the right-hand rule.

(5) The INS frame *O_g_X_g_Y_g_Z_g_* (*g-frame*). The three axes are in accordance with the sensitive directions of the gyro triad and the accelerometer triad of the INS, and they complete the right-handed frame.

### 2.2. Reference Frame Conversion

As previously described, the inertial and navigation frames are the reference frames of the IHDST and INS, respectively. Therefore, the reference frame of the IHDST should be converted from the *i-frame* to the *n-frame* before an attitude accuracy assessment of the INS. The reference frame conversion can be divided into two steps, namely, the first step from the *i-frame* to the *e-frame* and the second step from the *e-frame* to the *n-frame*.

#### 2.2.1. Conversion from the *i-frame* to the *e-frame*

**Figure 2:**
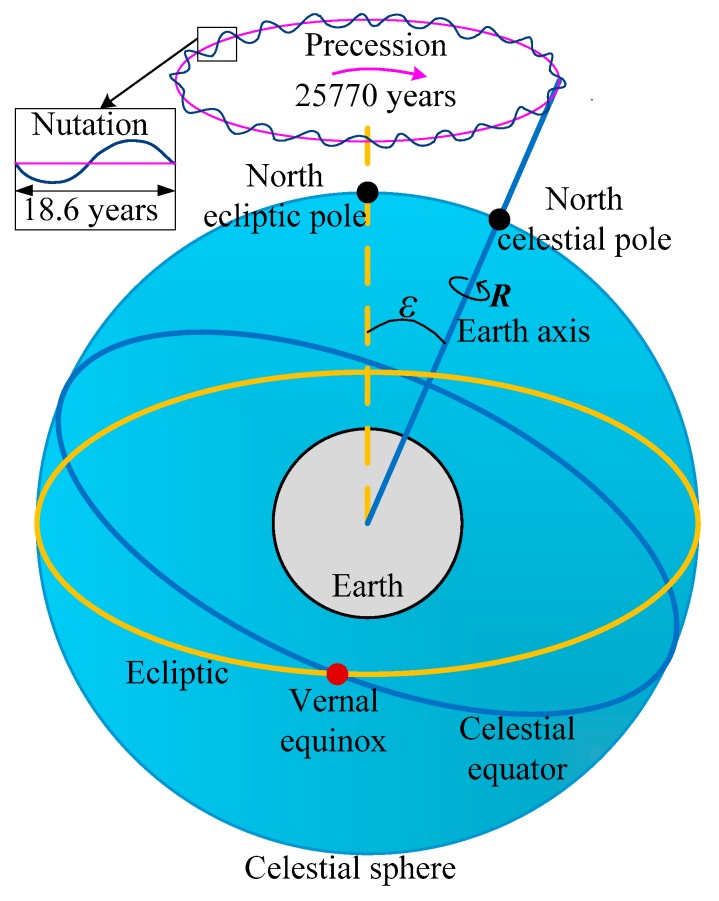
Motion of the earth’s axis.

In theory, the conversion from the *i-frame* to the *e-frame* can be realized merely depending on the earth’s rotation from the epoch of J2000.0 to the observation time *t*. However, besides the rotation movement of the earth, its rotation axis is also moving constantly in the *i-frame* due to the influence of other celestial bodies (e.g., the sun and the moon) as well as the irregularity of the earth itself [30]. Figure 2 shows the motion of the earth’s axis. Firstly, the north celestial pole revolves around the north ecliptic pole clockwise with a radius of the obliquity *ε* due to the mutual motion of the equatorial plane and the ecliptic plane, and the rotation period is about 25,770 years. Given that, a small west movement about 50.290˝ of the vernal equinox is generated every year. This movement is referred to as the precession. Besides that, the north celestial pole also has a small periodic elliptical swinging mainly because of the gravitation of the sun, the moon, and other celestial bodies to the earth. This movement is referred to as the nutation, and its period is about 18.6 years. Lastly, the polar motion of the earth also exists, but this kind of motion is quite small and thus can be ignored in this paper.

In summary, the earth’s rotation as well as the precession and nutation should be considered and compensated for, thus accurately realizing the conversion from the *i-frame* at the epoch of J2000.0 to the *e-frame* at the observation time *t*. Firstly, the mean celestial coordinate frame *O_e_X_im_Y_im_Z_im_* (*i_m_-frame*) is obtained when the precession is compensated to the *i-frame*. The celestial pole, the celestial equator, and the vernal equinox corresponding to the *i_m_-frame* are referred to as the mean celestial pole, the mean celestial equator, and the mean vernal equinox, respectively. Subsequently, the instantaneous celestial coordinate frame *O_e_X_it_Y_it_Z_it_* (*i_t_-frame*) at the observation time *t* can be obtained when the nutation is compensated to the *i_m_-frame*. The *i_t_-frame* is established by using the instantaneous north celestial pole as the base point, the instantaneous celestial equator as the base circle, and the instantaneous vernal equinox as the principal point, respectively. Lastly, the *e-frame* at the observation time *t* can be obtained when the earth’s rotation is compensated to the *i_t_-frame*.

Let *P*(*t*), *N*(*t*), and *R*(*t*) be the precession, nutation, and earth rotation matrices, respectively, and then the preceding frame conversions can be expressed as follows [30]:

(1) $$\left[\begin{array}{c} X_{im}\\ Y_{im}\\ Z_{im} \end{array}\right]=P(t)\left[\begin{array}{c} X_{i}\\ Y_{i}\\ Z_{i} \end{array}\right],\;\left[\begin{array}{c} X_{it}\\ Y_{it}\\ Z_{it} \end{array}\right]=N(t)\left[\begin{array}{c} X_{im}\\ Y_{im}\\ Z_{im} \end{array}\right],\;\left[\begin{array}{c} X_{e}\\ Y_{e}\\ Z_{e} \end{array}\right]=R(t)\left[\begin{array}{c} X_{it}\\ Y_{it}\\ Z_{it} \end{array}\right].$$

##### *1) Precession Matrix* 

The precession matrix in Equation (1) can be entirely determined by the three rotation matrices, and its expression is as follows:

(2) $$P(t)=R_{Z}(-z_{A})\cdot R_{Y}(\theta_{A})\cdot R_{Z}(-\zeta_{A}),$$

where *ζ_A_*, *θ_A_*, and *z_A_* are the precession parameters of the IAU2000 precession model [30], and *R_Z_* and *R_Y_* are the matrices rotated around the *Z*- and *Y*-axes, respectively. Let *δ* be the rotation angle, and then the matrices rotated around the *X*-, *Y*-, and *Z*-axes can be expressed as follows:

(3) $$\begin{array}{l} R_{X}(\delta )=\left[\begin{array}{ccc} 1 & 0 & 0\\ 0 & \cos(\delta ) & \sin(\delta )\\ 0 & -\sin(\delta ) & \cos(\delta ) \end{array}\right],\;R_{Y}(\delta )=\left[\begin{array}{ccc} \cos(\delta ) & 0 & -\sin(\delta )\\ 0 & 1 & 0\\ \sin(\delta ) & 0 & \cos(\delta ) \end{array}\right],\\ R_{Z}(\delta )=\left[\begin{array}{ccc} \cos(\delta ) & \sin(\delta ) & 0\\ -\sin(\delta ) & \cos(\delta ) & 0\\ 0 & 0 & 1 \end{array}\right]. \end{array}$$

##### *2) Nutation Matrix* 

Similarly, the nutation matrix *N*(*t*) can also be entirely determined by the three rotation matrices, and its expression is as follows [30]:

(4) $$N(t)=R_{X}(-\varepsilon -\Delta \varepsilon )\cdot R_{Z}(-\Delta \psi )\cdot R_{X}(\varepsilon ),$$

where *ε*, Δ*ψ*, and Δ*ε* are the obliquity, the longitude nutation, and the obliquity nutation in the IAU2000B nutation model, respectively [30,31].

##### *3) Earth Rotation Matrix* 

The earth rotation matrix *R*(*t*) is entirely determined by the Greenwich hour angle of the instantaneous vernal equinox (namely, *β_G_*), and its expression is as follows [30]:

(5) $$R(t)=R_{Z}(\beta_{G}).$$

In summary, the conversion from the *i-frame* at the epoch of J2000.0 to the *e-frame* at the observation time *t* can be realized in three steps according to Equation (1), and the total conversion expression is as follows:

(6) $$\left[\begin{array}{c} X_{e}\\ Y_{e}\\ Z_{e} \end{array}\right]=C_{i}^{e}(t)\left[\begin{array}{c} X_{i}\\ Y_{i}\\ Z_{i} \end{array}\right],\;C_{i}^{e}(t)=R(t)N(t)P(t),$$

where $C_{i}^{e}(t)$ is the total rotation matrix from the *i-frame* to the *e-frame*, and *P*(*t*), *N*(*t*), and *R*(*t*) are determined according to Equations (2)–(5).

#### 2.2.2. Conversion from the *e-frame* to the *n-frame*

As shown in Figure 1, the conversion from the *e-frame* to the *n-frame* can be realized merely depending on the longitude (*λ*) and latitude (*φ*) of the IHDST and INS at the observation time *t*. The corresponding conversion relation can be expressed as

(7) $$\left[\begin{array}{c} X_{n}\\ Y_{n}\\ Z_{n} \end{array}\right]=C_{e}^{n}(t)\left[\begin{array}{c} X_{e}\\ Y_{e}\\ Z_{e} \end{array}\right],$$

where $C_{e}^{n}(t)$ is the rotation matrix from the *e-frame* to the *n-frame*, and its expression, which is entirely determined by the three rotation matrices, can be written as

(8) $$\begin{array}{ll} C_{e}^{n}(t) & =R_{Y}(180{}^{\circ})\cdot R_{Y}(90{}^{\circ}-\varphi )\cdot R_{Z}(\lambda )\\ & =\left[\begin{array}{ccc} -\sin\varphi \cos\lambda & -\sin\varphi \sin\lambda & \cos\varphi \\ -\sin\lambda & \cos\lambda & 0\\ -\cos\varphi \cos\lambda & -\cos\varphi \sin\lambda & -\sin\varphi \end{array}\right]. \end{array}$$

According to Equations (6) and (7), the total conversion from the *i-frame* at the epoch of J2000.0 to the *n-frame* at the observation time *t* can be expressed as

(9) $$\left[\begin{array}{c} X_{n}\\ Y_{n}\\ Z_{n} \end{array}\right]=C_{e}^{n}(t)C_{i}^{e}(t)\left[\begin{array}{c} X_{i}\\ Y_{i}\\ Z_{i} \end{array}\right].$$

Let $Q_{i}^{s}(t)$ be the original attitude matrix of the IHDST with respect to the *i-frame* at the observation time *t*. Then, the conversion attitude matrix $Q_{n}^{s}(t)$ of the IHDST with respect to the *n-frame* can be obtained according to Equation (9). At this time, the reference frame of the IHDST has been converted to be consistent with that of the INS, and the corresponding conversion can be expressed as

(10) $$Q_{n}^{s}(t)=Q_{i}^{s}(t)C_{e}^{i}(t)C_{n}^{e}(t)=Q_{i}^{s}(t)\cdot {\left[C_{e}^{n}(t)C_{i}^{e}(t)\right]}^{T}.$$

## 3. Decoupling Estimation of the Systematic Coordinate Errors of an INS and IHDST

As mentioned earlier, the reference frame of the IHDST has been converted from the *i-frame* to the *n-frame* based on Equation (10), and the original attitude matrix $Q_{i}^{s}(t)$ of the IHDST has been accordingly transformed into the attitude matrix $Q_{n}^{s}(t)$ with respect to the *n-frame*. Meanwhile, the reference frame of the INS is the *n-frame*, and thus its original attitude matrix is $Q_{n}^{g}(t)$. In theory, the attitude matrix $Q_{n}^{s}(t)$ of the IHDST can be directly used as the benchmark to assess the accuracy of the attitude matrix $Q_{n}^{g}(t)$ of the INS. However, the actual coordinate frames of the IHDST and INS (i.e., the *s-frame* and the *g-frame*) cannot be exactly the same, and there always exists an installation error between the two. The installation error is the systematic error, and it must be compensated for, thus improving the assessment accuracy. Furthermore, according to the characteristics of the INS, its attitude error is mainly comprised of a misalignment error and an inertial instrument error. The former is the systematic error, and it should be accurately estimated and compensated for. The above two systematic coordinate errors severely decrease the accuracy of the assessment method, and their effects are coupled. Given that, a high-accuracy decoupling estimation method of the above two systematic coordinate errors based on CLS is proposed in this paper. The decoupling estimation model of the above two errors is first established, and then the CLS-based optimization method is utilized to estimate them accurately. Finally, the attitude accuracy of the INS can be assessed quantitatively after compensating for the above two errors.

### 3.1. Decoupling Estimation Model

Let $B_{s}^{g}$ be the installation error matrix from the *s-frame* to the *g-frame*. Then, the truth matrix ${\tilde{Q}}_{n}^{g}(t)$ of an INS can be derived from the attitude matrix $Q_{n}^{s}(t)$ of the IHDST as follows:

(11) $${\tilde{Q}}_{n}^{g}(t)=B_{s}^{g}Q_{n}^{s}(t).$$

Since the calculation of the attitude matrix is relatively complicated, it is necessary to transform the attitude matrix into its entirely equivalent attitude quaternion, thus simplifying the related intermediate calculation. Given that, the truth matrix ${\tilde{Q}}_{n}^{g}(t)$ is transformed into the truth quaternion ${\tilde{q}}_{n}^{g}(t)$, the original attitude matrix $Q_{n}^{g}(t)$ of the INS is transformed into the original attitude quaternion $q_{n}^{g}(t)$, and the installation error matrix $B_{s}^{g}$ is transformed into the installation error quaternion $b_{s}^{g}$, respectively. On this basis, Equation (11) can be converted to its quaternionic expression:

(12) $${\tilde{q}}_{n}^{g}(t)=q_{n}^{s}(t)⊗b_{s}^{g},$$

where “⊗” represents quaternionic multiplication. If the measurement moment *t* is selected as *t* = *t_i_* (*i* = 1, 2, 3, …, *N*), the corresponding loss function *L_a_* can be expressed as

(13) $$L_{a}=\sum_{i=1}^{N}{\left|q_{n}^{g}(t_{i})-{\tilde{q}}_{n}^{g}(t_{i})\right|}^{2}=\sum_{i=1}^{N}{\left|q_{n}^{g}(t_{i})-q_{n}^{s}(t_{i})⊗b_{s}^{g}\right|}^{2}.$$

**Figure 3:**
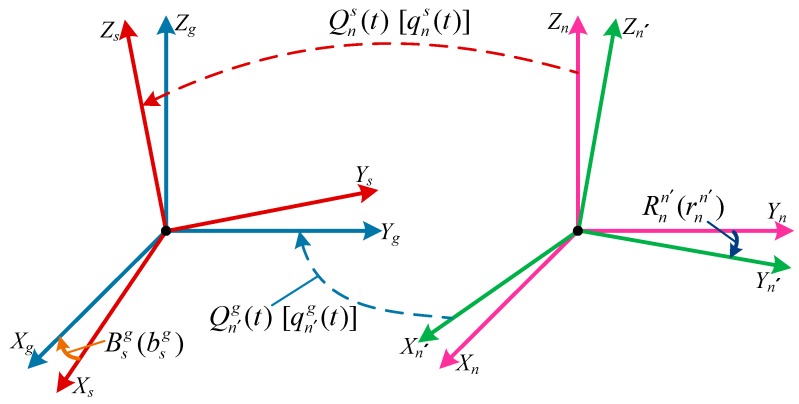
Complete transformation relations of coordinate frames.

In theory, the optimal estimation ${\hat{b}}_{s}^{g}$ of the installation error quaternion can be obtained when *L_a_* takes its minimum value. However, besides the installation error between the INS and the IHDST, there also exists the misalignment error of the INS, and the effects of the above two errors are mutually coupled. The optimal estimation ${\hat{b}}_{s}^{g}$ cannot be obtained any more when merely depending on the loss function *L_a_* in Equation (13). Figure 3 shows the complete transformation relations of the coordinate frames. The original attitude quaternion of the INS is not $q_{n}^{g}(t)$, but $q_{n^{\prime }}^{g}(t)$, where *n* and *n*´ represent the ideal navigation frame (*n-frame*) and the real navigation frame (*n*´*-frame*), respectively, and the misalignment error quaternion $r_{n}^{n^{\prime }}$ (its entirely equivalent matrix is $R_{n}^{n^{\prime }}$) always exists between the two frames.

After compensating for misalignment error, the attitude quaternion of the INS can be expressed as

(14) $$q_{n}^{g}(t)=r_{n}^{n^{\prime }}⊗q_{n^{\prime }}^{g}(t).$$

By substituting Equation (14) into (13), the complete loss function *L_b_*, including the misalignment error quaternion $r_{n}^{n^{\prime }}$ and the installation error quaternion $b_{s}^{g}$, can be expressed as

(15) $$L_{b}=\sum_{i=1}^{N}{\left|q_{n}^{g}(t_{i})-{\tilde{q}}_{n}^{g}(t_{i})\right|}^{2}=\sum_{i=1}^{N}{\left|r_{n}^{n^{\prime }}⊗q_{n^{\prime }}^{g}(t_{i})-q_{n}^{s}(t_{i})⊗b_{s}^{g}\right|}^{2},$$

where

(16) $$\begin{array}{l} q_{n^{\prime }}^{g}(t_{i})={\left[\begin{array}{cccc} y_{i0} & y_{i1} & y_{i2} & y_{i3} \end{array}\right]}^{T},q_{n}^{s}(t_{i})={\left[\begin{array}{cccc} x_{i0} & x_{i1} & x_{i2} & x_{i3} \end{array}\right]}^{T},\\ r_{n}^{n^{\prime }}={\left[\begin{array}{cccc} r_{0} & r_{1} & r_{2} & r_{3} \end{array}\right]}^{T},b_{s}^{g}={\left[\begin{array}{cccc} b_{0} & b_{1} & b_{2} & b_{3} \end{array}\right]}^{T},i=1,\;2,\;3,\;\ldots ,\;N, \end{array}$$

and

(17) $$r_{0}^{2}+r_{1}^{2}+r_{2}^{2}+r_{3}^{2}=1,b_{0}^{2}+b_{1}^{2}+b_{2}^{2}+b_{3}^{2}=1.$$

Equations (15)–(17) represent the decoupling estimation model of the installation and misalignment errors.

### 3.2. CLS-Based Optimization Method

Equations (15) and (17), which are the objective function and the equality constraints respectively, constitute a typical CLS problem. By means of equivalent transformation, the equality constraints in Equation (17) can be rewritten as

(18) $$\begin{array}{l} h_{1}=r_{0}^{2}+r_{1}^{2}+r_{2}^{2}+r_{3}^{2}-1=0,\\ h_{2}=\;b_{0}^{2}+b_{1}^{2}+b_{2}^{2}+b_{3}^{2}-1=0. \end{array}$$

The objective function *L_b_* in Equation (15) is calculated, and the result is equal to the square sum of the quaternion deviations at *N* measurement moments. For the moment *t_i_*, the corresponding quaternion deviation can be expressed as

(19) $$\begin{array}{cc} \Delta q_{i} & =r_{n}^{n^{\prime }}⊗q_{n^{\prime }}^{g}(t_{i})-q_{n}^{s}(t_{i})⊗b_{s}^{g}\\ & \;=\left[\begin{array}{c} r_{0}y_{i0}-r_{1}y_{i1}-r_{2}y_{i2}-r_{3}y_{i3}-b_{0}x_{i0}+b_{1}x_{i1}+b_{2}x_{i2}+b_{3}x_{i3}\\ r_{1}y_{i0}+r_{0}y_{i1}-r_{3}y_{i2}+r_{2}y_{i3}-b_{1}x_{i0}-b_{0}x_{i1}-b_{3}x_{i2}+b_{2}x_{i3}\\ r_{2}y_{i0}+r_{3}y_{i1}+r_{0}y_{i2}-r_{1}y_{i3}-b_{2}x_{i0}+b_{3}x_{i1}-b_{0}x_{i2}-b_{1}x_{i3}\\ r_{3}y_{i0}+r_{2}y_{i1}+r_{1}y_{i2}+r_{0}y_{i3}-b_{3}x_{i0}-b_{2}x_{i1}+b_{1}x_{i2}-b_{0}x_{i3} \end{array}\right]. \end{array}$$

The above quaternion deviation Δ*q_i_* contains a total of four components, each of which consists of eight cross terms. When calculating the square sum of the 4 components of Δ*q_i_*, each component can generate 8 square terms and 28 cross-product terms, and thus 4 components totally generate 32 square terms and 112 cross-product terms. The sum Σ*_i_*_,_*_sq_* of all the 32 square terms can be derived as follows:

(20) $$\begin{array}{ll} \Sigma_{i,sq} & =(r_{0}^{2}+r_{1}^{2}+r_{2}^{2}+r_{3}^{2})\cdot (y_{i0}^{2}+y_{i1}^{2}+y_{i2}^{2}+y_{i3}^{2})\\ & \;+(b_{0}^{2}+b_{1}^{2}+b_{2}^{2}+b_{3}^{2})\cdot (x_{i0}^{2}+x_{i1}^{2}+x_{i2}^{2}+x_{i3}^{2})\\ & \;=1\times 1+1\times 1=2. \end{array}$$

When calculating the sum Σ*_i_*_,_*_cro_* of all the 112 cross-product terms and combining the similar terms, the expression of the ultimate result can be simplified as

(21) $$\Sigma_{i,cro}=\Sigma_{i,cro1}+\Sigma_{i,cro2}+\Sigma_{i,cro3}+\Sigma_{i,cro4},$$

where

(22) $$\begin{array}{l} \Sigma_{i,cro1}=2b_{0}r_{0}\cdot (-d_{i00}-d_{i11}-d_{i22}-d_{i33})+2b_{0}r_{1}\cdot (d_{i01}-d_{i10}+d_{i23}-d_{i32})\\ +2b_{0}r_{2}\cdot (d_{i02}-d_{i13}-d_{i20}+d_{i31})+2b_{0}r_{3}\cdot (d_{i03}+d_{i12}-d_{i21}-d_{i30}), \end{array}$$

(23) $$\begin{array}{l} \Sigma_{i,cro2}=2b_{1}r_{0}\cdot (d_{i10}-d_{i01}-d_{i32}+d_{i23})+2b_{1}r_{1}\cdot (-d_{i11}-d_{i00}+d_{i33}+d_{i22})\\ +2b_{1}r_{2}\cdot (-d_{i12}-d_{i03}-d_{i30}-d_{i21})+2b_{1}r_{3}\cdot (-d_{i13}+d_{i02}-d_{i31}+d_{i20}), \end{array}$$

(24) $$\begin{array}{l} \Sigma_{i,cro3}=2b_{2}r_{0}\cdot (d_{i20}+d_{i31}-d_{i02}-d_{i13})+2b_{2}r_{1}\cdot (-d_{i21}+d_{i30}+d_{i03}-d_{i12})\\ +2b_{2}r_{2}\cdot (-d_{i22}+d_{i33}-d_{i00}+d_{i11})+2b_{2}r_{3}\cdot (-d_{i23}-d_{i32}-d_{i01}-d_{i10}), \end{array}$$

(25) $$\begin{array}{l} \Sigma_{i,cro4}=2b_{3}r_{0}\cdot (d_{i30}-d_{i21}+d_{i12}-d_{i03})+2b_{3}r_{1}\cdot (-d_{i31}-d_{i20}-d_{i13}-d_{i02})\\ +2b_{3}r_{2}\cdot (-d_{i32}-d_{i23}+d_{i10}+d_{i01})+2b_{3}r_{3}\cdot (-d_{i33}+d_{i22}+d_{i11}-d_{i00}), \end{array}$$

and

(26) $$d_{ijk}=x_{ij}y_{ik},\;j=0,1,2,3,\;k=0,1,2,3.$$

When the above results at moment *t_i_* are extended to all the *N* measurement moments, the expression of the objective function *L_b_* in Equation (15) can be derived as follows:

(27) $$\begin{array}{ll} L_{b} & =\sum_{i=1}^{N}{\left|r_{n}^{n^{\prime }}⊗q_{n^{\prime }}^{g}(t_{i})-q_{n}^{s}(t_{i})⊗b_{s}^{g}\right|}^{2}=\sum_{i=1}^{N}\left(\Sigma_{i,sq}+\Sigma_{i,cro}\right)\\ & =2N+\sum_{i=1}^{N}\left(\Sigma_{i,cro1}+\Sigma_{i,cro2}+\Sigma_{i,cro3}+\Sigma_{i,cro4}\right), \end{array}$$

where Σ*_i_*_,*cro*1_, Σ*_i_*_,*cro*2_, Σ*_i_*_,*cro*3_, and Σ*_i_*_,*cro*4_ are determined by Equations (22)–(26).

After the preceding calculations, the optimal estimations ${\hat{b}}_{s}^{g}$ and ${\hat{r}}_{n}^{n^{\prime }}$ can be obtained simultaneously when *L_b_* in Equation (27) takes its minimum value under the condition of Equation (18). Defining the coefficient vector as [*λ*_1_ *λ*_2_]^T^, the Lagrange function can be constructed as [32]

(28) $$l(b_{j},r_{j},\lambda_{k})=L_{b}+\lambda_{1}\cdot h_{1}+\lambda_{2}\cdot h_{2},\;j=0,1,2,3,\;k=1,2.$$

Therefore, ${\hat{b}}_{s}^{g}$ and ${\hat{r}}_{n}^{n^{\prime }}$ should satisfy the following Lagrange condition [32]:

(29) $$\frac{\partial l}{\partial b_{j}}=0,\;\frac{\partial l}{\partial r_{j}}=0,\;\frac{\partial l}{\partial \lambda_{k}}=0,\;j=0,1,2,3,\;k=1,2.$$

Equation (29) contains a total of 10 sub equations. The last two are obtained by solving the partial derivatives of a Lagrange function *l* relative to *λ_k_* (*k* = 1, 2), and they are exactly the same as Equation (18). The other eight are the partial derivatives relative to *b_j_* and *r_j_* (*j* = 0, 1, 2, 3), and they can be reorganized as follows:

(30) $$V\cdot R=-\lambda_{1}\cdot B,$$

(31) $$V^{T}\cdot B=-\lambda_{2}\cdot R,$$

whose matrix component forms are expressed as

(32) $$\sum_{i=1}^{N}\left[\begin{array}{llll} v_{i11} & v_{i12} & v_{i13} & v_{i14}\\ v_{i21} & v_{i22} & v_{i23} & v_{i24}\\ v_{i31} & v_{i32} & v_{i33} & v_{i34}\\ v_{i41} & v_{i42} & v_{i43} & v_{i44} \end{array}\right]\cdot \left[\begin{array}{c} r_{0}\\ r_{1}\\ r_{2}\\ r_{3} \end{array}\right]=-\lambda_{1}\left[\begin{array}{c} b_{0}\\ b_{1}\\ b_{2}\\ b_{3} \end{array}\right],$$

(33) $$\sum_{i=1}^{N}{\left[\begin{array}{llll} v_{i11} & v_{i12} & v_{i13} & v_{i14}\\ v_{i21} & v_{i22} & v_{i23} & v_{i24}\\ v_{i31} & v_{i32} & v_{i33} & v_{i34}\\ v_{i41} & v_{i42} & v_{i43} & v_{i44} \end{array}\right]}^{T}\cdot \left[\begin{array}{c} b_{0}\\ b_{1}\\ b_{2}\\ b_{3} \end{array}\right]=-\lambda_{2}\left[\begin{array}{c} r_{0}\\ r_{1}\\ r_{2}\\ r_{3} \end{array}\right],$$

where

(34) $$\begin{array}{l} v_{i11}=-d_{i00}-d_{i11}-d_{i22}-d_{i33},v_{i12}=d_{i01}-d_{i10}+d_{i23}-d_{i32},\\ v_{i13}=d_{i02}-d_{i13}-d_{i20}+d_{i31},v_{i14}=d_{i03}+d_{i12}-d_{i21}-d_{i30},\\ v_{i21}=d_{i10}-d_{i01}-d_{i32}+d_{i23},v_{i22}=-d_{i11}-d_{i00}+d_{i33}+d_{i22},\\ v_{i23}=-d_{i12}-d_{i03}-d_{i30}-d_{i21},v_{i24}=-d_{i13}+d_{i02}-d_{i31}+d_{i20},\\ v_{i31}=d_{i20}+d_{i31}-d_{i02}-d_{i13},v_{i32}=-d_{i21}+d_{i30}+d_{i03}-d_{i12},\\ v_{i33}=-d_{i22}+d_{i33}-d_{i00}+d_{i11},v_{i34}=-d_{i23}-d_{i32}-d_{i01}-d_{i10},\\ v_{i41}=d_{i30}-d_{i21}+d_{i12}-d_{i03},v_{i42}=-d_{i31}-d_{i20}-d_{i13}-d_{i02},\\ v_{i43}=-d_{i32}-d_{i23}+d_{i10}+d_{i01},v_{i44}=-d_{i33}+d_{i22}+d_{i11}-d_{i00}. \end{array}$$

In theory, ${\hat{b}}_{s}^{g}$ and ${\hat{r}}_{n}^{n^{\prime }}$, as well as [*λ*_1_ *λ*_2_]^T^, can be entirely determined by combining Equations (18), (30), and (31). However, the above equations are nonlinear, thus resulting in the solution process being quite complicated. Given that, proper equivalent transformations are conducted on Equations (30) and (31) for a calculation simplification. By multiplying the coefficient *λ*_2_ on both sides of Equation (30) and then substituting Equation (31) into it, the result can be derived as

(35) $$-\lambda_{1}\cdot \lambda_{2}\cdot B=V\cdot \lambda_{2}\cdot R=V\cdot (-V^{T}\cdot B)=-(V\cdot V^{T})\cdot B,$$

namely,

(36) $$P\cdot B=(\lambda_{1}\lambda_{2})\cdot B,\;P=V\cdot V^{T}.$$

Similarly, when multiplying the coefficient *λ*_1_ on both sides of Equation (31) and then substituting Equation (30) into it, the result can be derived as

(37) $$Q\cdot R=(\lambda_{1}\lambda_{2})\cdot R,\;Q=V^{T}\cdot V.$$

The norms of *B* and *R* are equal to 1, and thus they are both four-dimensional nonzero vectors. According to Equations (36) and (37), *B* and *R* can be considered as the eigenvectors, which belong to the eigenvalue *λ*_1_*λ*_2_ of the matrices *P* and *Q*, respectively. Since the Lagrange condition described in Equation (29) is only necessary, not sufficient, all of the four eigenvectors of *P* and *Q* should be solved and then used to calculate the objective function *L_b_* in Equation (27). The optimal estimations ${\hat{b}}_{s}^{g}$ and ${\hat{r}}_{n}^{n^{\prime }}$ can be obtained by the eigenvectors B^*^ and R^*^, which make *L_b_* take its minimum value. The expressions are as follows:

(38) $${\hat{b}}_{s}^{g}=B^{*}={\left[\begin{array}{cccc} b_{0}^{*} & b_{1}^{*} & b_{2}^{*} & b_{3}^{*} \end{array}\right]}^{T},\;{\hat{r}}_{n}^{n^{\prime }}=R^{*}={\left[\begin{array}{cccc} r_{0}^{*} & r_{1}^{*} & r_{2}^{*} & r_{3}^{*} \end{array}\right]}^{T}.$$

As previously described, ${\hat{b}}_{s}^{g}$ and ${\hat{r}}_{n}^{n^{\prime }}$ can be transformed into their entirely equivalent attitude matrices ${\hat{B}}_{s}^{g}$ and ${\hat{R}}_{n}^{n^{\prime }}$, respectively. After compensating for the installation error, the truth matrix ${\tilde{Q}}_{n}^{g}(t)$ of the INS with respect to the *n-frame* can be derived from the attitude matrix $Q_{n}^{s}(t)$ of the IHDST, which is shown in Equation (11). Meanwhile, after compensating for the misalignment error, the measurement attitude matrix ${\bar{Q}}_{n}^{g}(t)$ of the INS can also be expressed as

(39) $${\bar{Q}}_{n}^{g}(t)=Q_{n^{\prime }}^{g}(t)\cdot {\hat{R}}_{n}^{n^{\prime }},$$

where $Q_{n^{\prime }}^{g}(t)$ is the original attitude matrix of the INS.

Although the attitude matrix or quaternion can clearly represent the rotation relation, they do not have any dimension, and each component does not have any independent physical meaning either. Given that, the ultimate output attitude parameters are transformed into the three-axis Euler angles in degrees (°), which are entirely equivalent to the attitude matrix or quaternion. In this study, the three-rotation order is defined as *Z*-*Y*-*X* (i.e., 3-2-1), and the corresponding Euler angles are referred to as yaw (*φ*), pitch (*θ*), and roll (*γ*), respectively.

Let the truth Euler angles of an INS according to Equation (11) and the measurement Euler angles of the INS according to Equation (39) be $\left({\tilde{\gamma }}_{s},{\tilde{\theta }}_{s},{\tilde{\varphi }}_{s}\right)$ and $\left({\bar{r}}_{g},{\bar{\theta }}_{g},{\bar{\varphi }}_{g}\right)$, respectively. Then, the absolute measurement errors (AMEs) of the attitude parameters of the INS based on Euler angles can be expressed as follows:

(40) $$\Delta \gamma_{g}={\bar{r}}_{g}-{\tilde{\gamma }}_{s},\;\Delta \theta_{g}={\bar{\theta }}_{g}-{\tilde{\theta }}_{s},\;\Delta \varphi_{g}={\bar{\varphi }}_{g}-{\tilde{\varphi }}_{s}.$$

## 4. Experiments and Discussion

Both simulated and real experiments are conducted in this section to verify the feasibility and effectiveness of the proposed method.

### 4.1. Simulated Experiments

**Figure 4:**
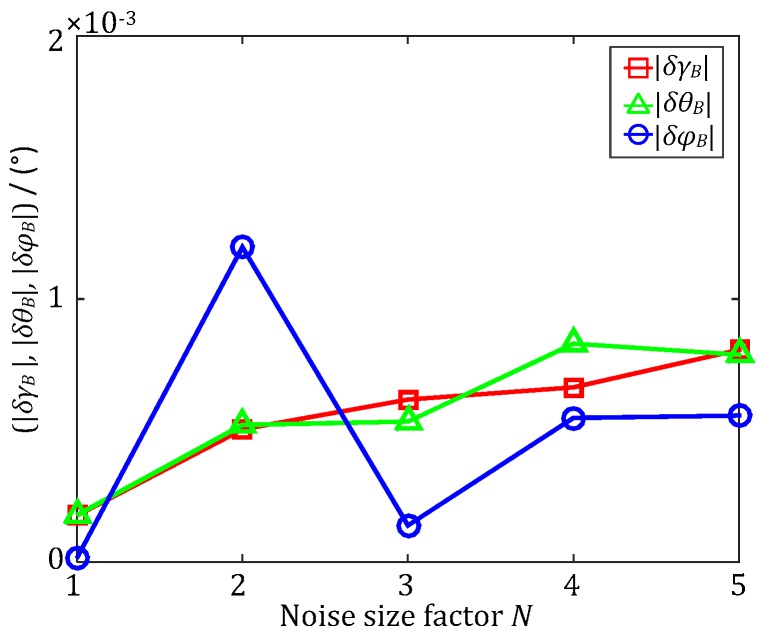
The absolute values of the estimation errors (|*δγ_B_*|, |*δθ_B_*|, |*δφ_B_*|) of the installation error.

**Figure 5:**
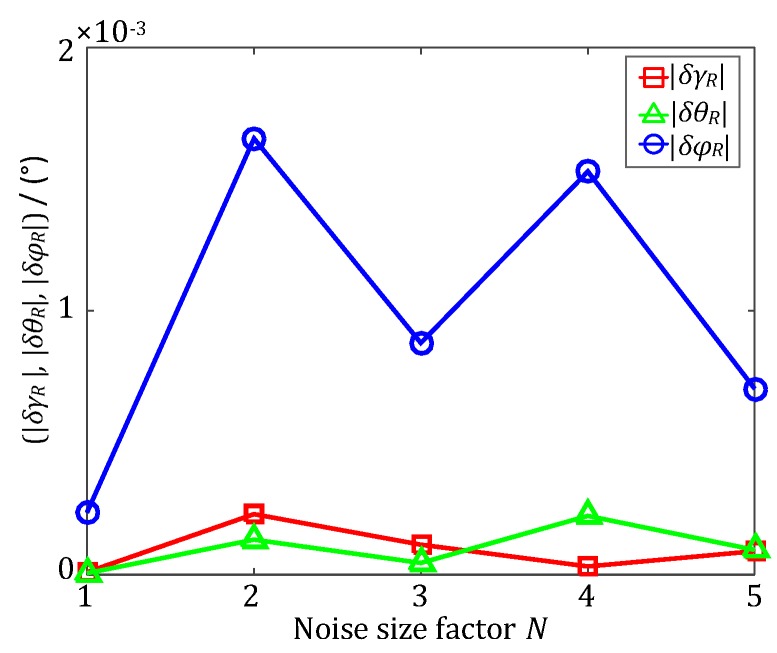
The absolute values of the estimation errors (|*δγ_R_*|, |*δθ_R_*|, |*δφ_R_*|) of the misalignment error.

In order to verify the accuracy performance of the decoupling estimation method for systematic coordinate errors proposed in this paper, simulated experiments are conducted in this section. Firstly, the truth Euler angles of the installation error (*γ_B_*, *θ_B_*, *φ_B_*) and the misalignment error (*γ_R_*, *θ_R_*, *φ_R_*) are randomly generated as (0.4572, −0.0146, 0.3003) and (−0.0782, 0.4157, 0.2922) in degrees (°), respectively. Then, the initial attitude $q_{n}^{s}(t_{0})$ of the IHDST is randomly generated, and the subsequent attitude $q_{n}^{s}(t)$ of the IHDST can be derived according to the preset motion condition. The sampling frequency of the IHDST is set as 25 Hz, and the total simulated time is set as 300 s. Subsequently, According to Equations (12) and (14), the attitude $q_{n^{\prime }}^{g}(t)$ of the INS can be determined on the basis of $q_{n}^{s}(t)$, (*γ_B_*, *θ_B_*, *φ_B_*) and (*γ_R_*, *θ_R_*, *φ_R_*). Lastly, Gaussian noises are added to the attitude data of the IHDST and the INS, respectively, to simulate the random error. The Gaussian noise of the IHDST is set to zero mean and 5×*N* arc-seconds (‘’) standard deviation in 1σ, while the Gaussian noise of the INS is set to zero mean and 0.01×*N* ° standard deviation in 1σ, where *N* is the Noise size factor, and it is set as 1, 2, 3, 4, 5, respectively. At this time, the proposed decoupling estimation method can be utilized to estimate the installation and misalignment errors. Each set of simulations is repeated five times, and the mean values of the five times are outputted as the ultimate estimation results. Figure 4 and Figure 5 show the absolute values of the estimation errors of the installation and misalignment errors, respectively, which are all less than 2×10^-3^ ° regardless of the size of the Gaussian noise.

### 4.2. Real Experiments

**Figure 6:**
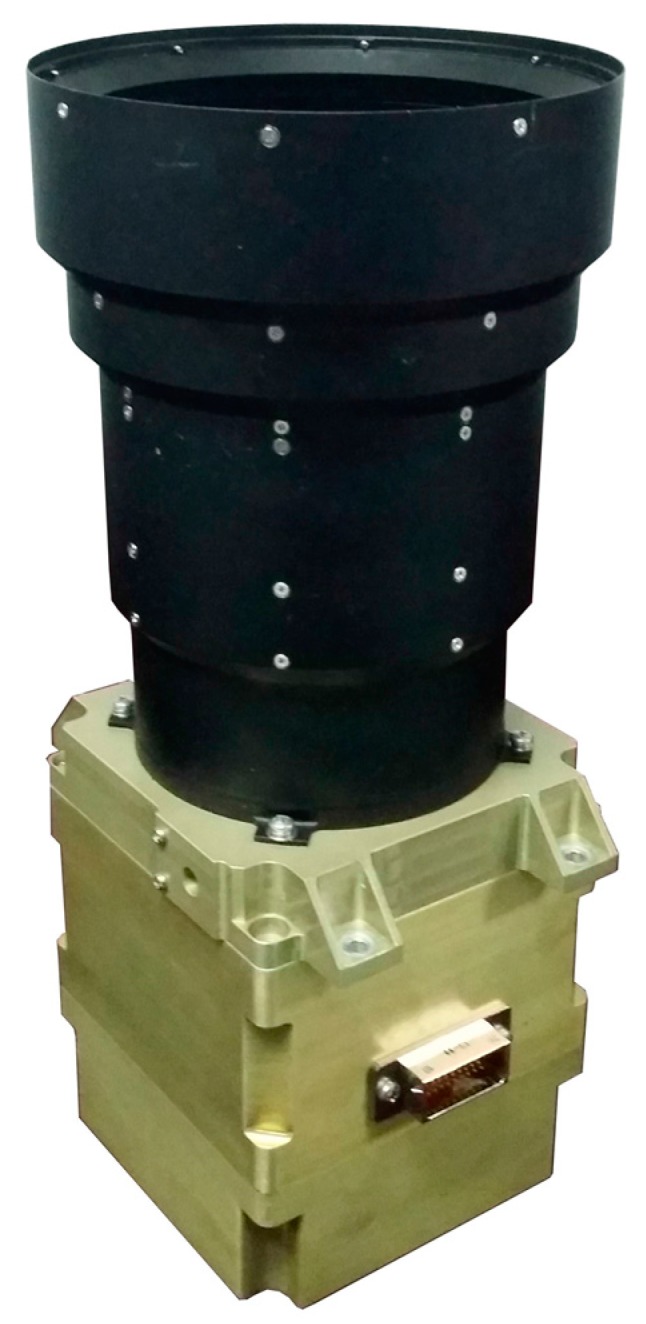
Self-developed IHDST.

In order to further validate the feasibility and effectiveness of the proposed method, real assessment experiments of attitude accuracy of an INS based on the IHDST, which is self-developed in this study, are conducted under an actual flight environment of an aircraft. Figure 6 shows the self-developed IHDST, and Table 1 lists its main performance specifications.

**Figure 7:**
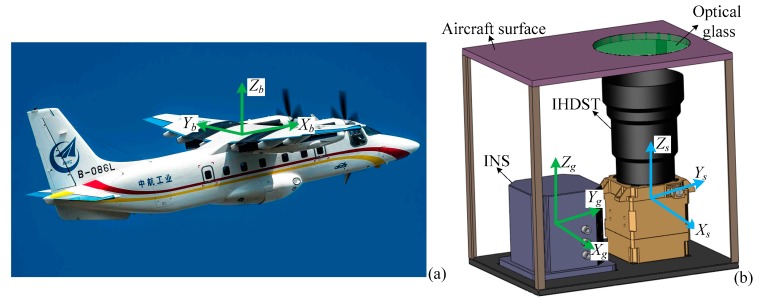
Experimental equipment. (**a**) Test aircraft; (**b**) Assessment setup mounted in the test aircraft.

Figure 7 shows all of the experimental equipment. The selected test aircraft is shown in Figure 7a, and the *X_b_*-, *Y_b_*-, and *Z_b_*-axes of its body frame (*b-frame*) are along the fuselage, wing, and vertical upward directions, respectively. The assessment setup, including the INS and IHDST, is stably mounted in the test aircraft, ensuring that the *s-frame* and *g-frame* are consistent with the *b-frame* as much as possible, which is shown in Figure 7b. The assessment experiments were conducted on a clear night without moonlight interference. After taking off, the test aircraft climbed up to a height of about 3000 meters (m) and then completed the flight of figure “8”, thus making the INS sufficiently convergent. Subsequently, the test aircraft climbed up to a height of about 4000 m and then completed various operations (i.e., level flight, sideward flight, and flight of figure “8”) according to the preset flight plan. Finally, the test aircraft fell back to the height of about 3000 m and maintained level flight for some time. The flight velocity of the test aircraft was about 250–320 kilometers per hour (km/h). These flight operations can make the assessment setup, including the INS and IHDST, experience a variety of maneuvering conditions, thus fully verifying the accuracy performance of the INS under real maneuvering environments.

**Figure 8:**
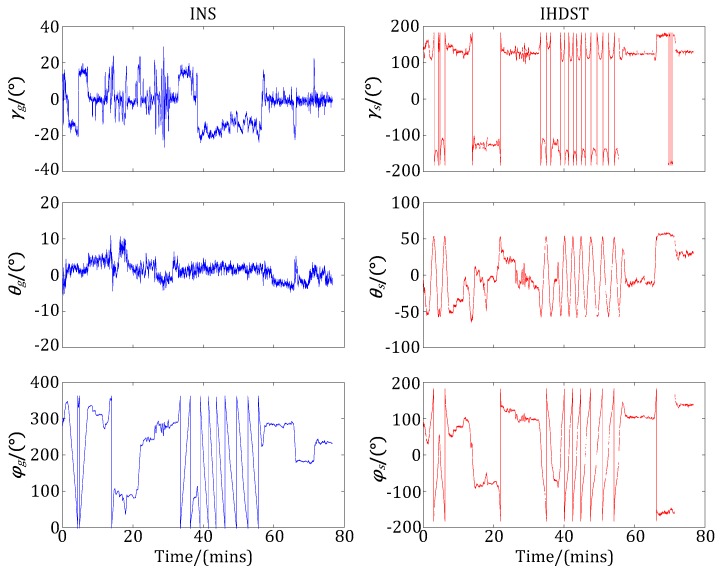
Raw three-axis Euler angles of the INS and IHDST.

**Figure 9:**
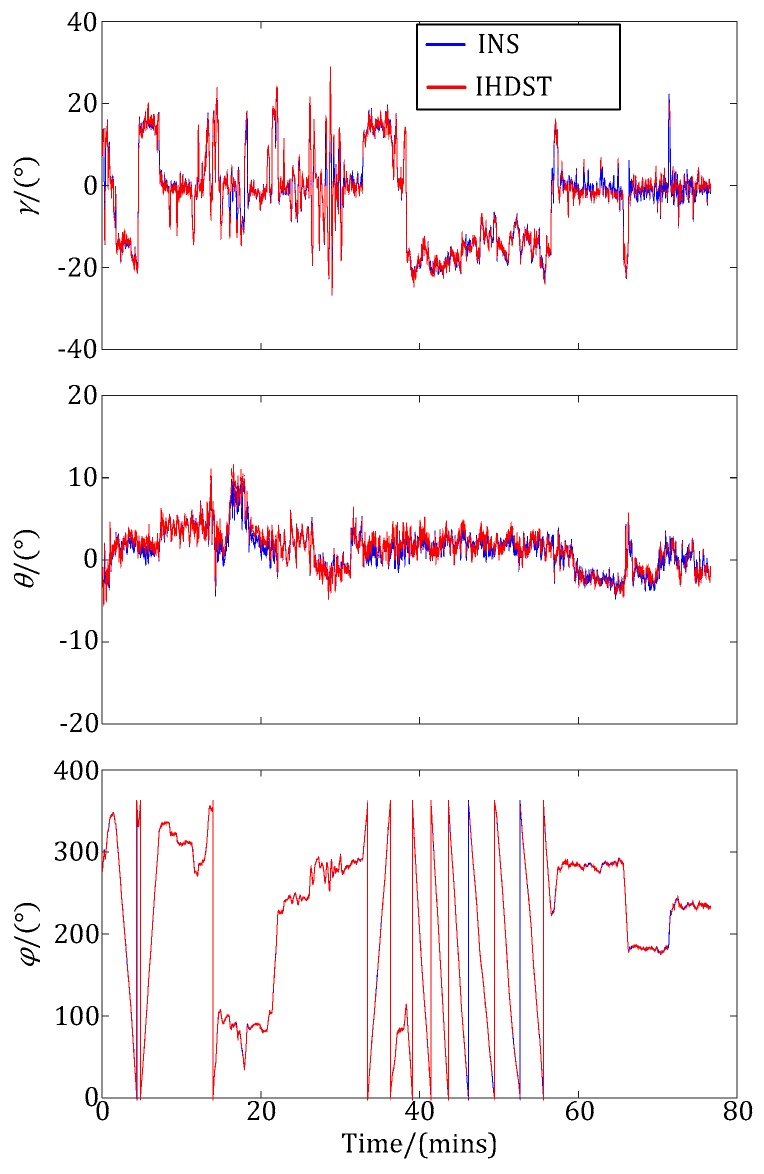
Converted data of the IHDST and raw data of the INS.

After the experiments, the test aircraft landed at the airport, and then the experimental attitude data of the INS and IHDST recorded in the memory was read out and analyzed. Figure 8 shows the raw three-axis Euler angles obtained by the INS and IHDST, respectively. (*γ_s_*, *θ_s_*, *φ_s_*) are the raw data of the IHDST with respect to the *i-frame*, while (*γ_g_*, *θ_g_*, *φ_g_*) are the raw data of the INS with respect to the *n-frame*. The correlation characteristic between the above two groups of data cannot be directly analyzed from Figure 8. Given that, the reference frame of the IHDST should be converted from the *i-frame* to the *n-frame* according to the preceding principle in this study. The converted data of the IHDST and the raw data of the INS can be plotted in the same figure, which is shown in Figure 9. After the conversion, the above two groups of data have a significant correlation characteristic in attitude variation.

**Figure 10:**
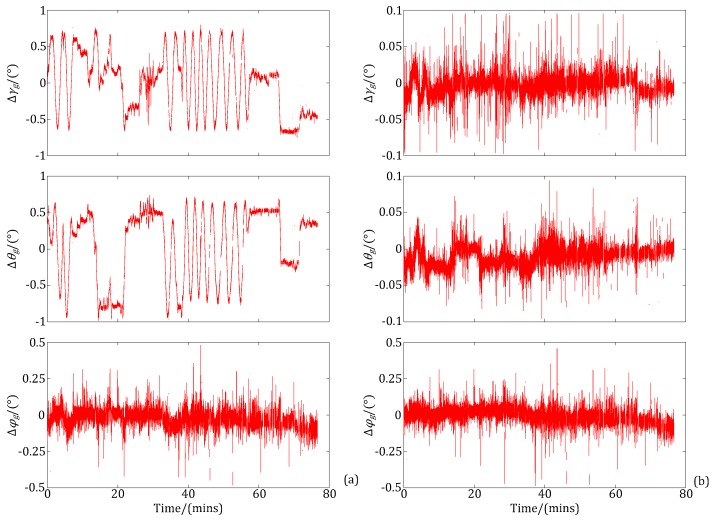
Absolute measurement errors (AMEs) of the Euler angles of the INS. (**a**) Before error estimation and compensation; (**b**) After error estimation and compensation.

As previously described, the IHDST can be used as the benchmark for an attitude accuracy assessment of the INS. The AMEs of Euler angles of the INS before and after the proposed decoupling estimation of and compensation for the systematic coordinate errors are shown in Figure 10a,b, respectively. In Figure 10a, the AMEs of roll (*γ*) and pitch (*θ*) of the INS show significant regular fluctuation characteristics. The fluctuation amplitude is relatively large, and it is about 1°. This makes it difficult to assess the attitude errors of the INS accurately. After the proposed decoupling estimation of and compensation for the systematic coordinate errors, the regular fluctuation has been eliminated effectively, and the AMEs of roll (*γ*) and pitch (*θ*) of the INS are reduced by about one order of magnitude, which is shown in Figure 10b.

**Figure 11:**
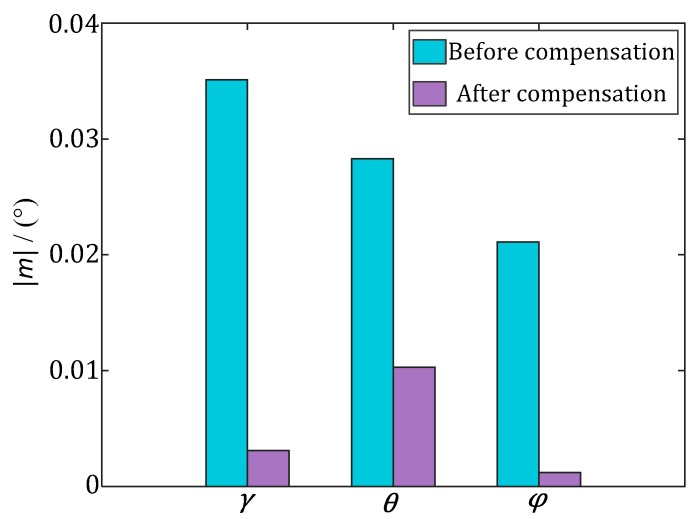
Absolute value of mean value (|*m*|) of the AMEs of the Euler angles of the INS.

**Figure 12:**
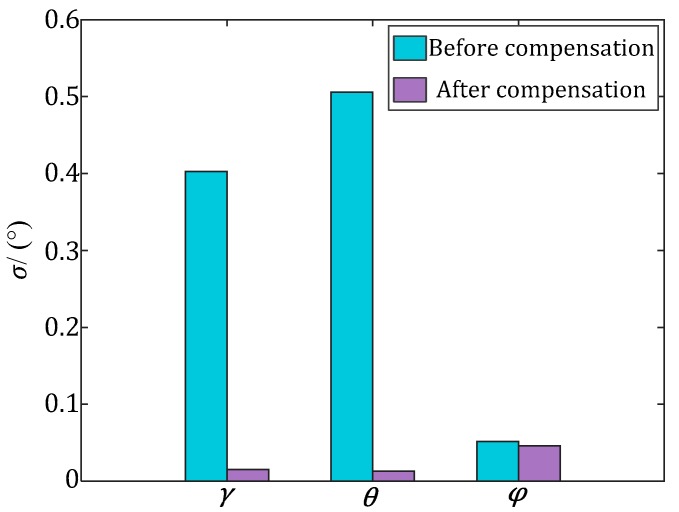
Standard deviation (*σ*) of the AMEs of the Euler angles of the INS.

The mean value and standard deviation of the AMEs of the INS in Figure 10 are shown in Figure 11 and Figure 12, respectively. After the estimation of and compensation for the above two errors, the mean value and standard deviation of the AMEs of the INS have been reduced significantly. For the roll (*γ*) and pitch (*θ*), the standard deviations are reduced about 30 times after error compensation, which means the errors are more concentrated. For the yaw (*φ*), the mean value is reduced about 15 times after error compensation, which means the error distribution is closer to the ideal zero. The corresponding optimal estimations of the installation and misalignment errors are listed in Table 2.

## 5. Conclusions

The attitude accuracy assessment of an INS based on the IHDST is particularly suitable for a real complex dynamic environment. The coupled systematic coordinate errors severely decrease the assessment accuracy. Given that, a high-accuracy decoupling estimation method for the above systematic coordinate errors based on CLS is proposed in this paper. This method only utilizes the attitude data of the INS and the IHDST to accurately estimate the above two errors without knowledge of the measurement models of the INS and IHDST. Both simulated and real experiments were conducted to verify the feasibility and effectiveness of the proposed method. The simulated results show that the absolute values of the estimation errors of the installation and misalignment errors are all less than 2×10^−3^ ° regardless of the size of the Gaussian noise. Furthermore, the real assessment experiments of the attitude accuracy of an INS are conducted under the actual flight environment of an aircraft. The IHDST is used as the benchmark for the attitude accuracy assessment of the INS. Before error compensation, the AMEs of the roll (*γ*) and pitch (*θ*) of the INS show significant regular fluctuation characteristics and the amplitude is about 1°. By contrast, after error compensation, the regular fluctuation has been eliminated effectively, and the standard deviations of the roll (*γ*) and pitch (*θ*) are reduced about 30 times, which means the errors are more concentrated. For the yaw (*φ*), the mean value is reduced about 15 times after error compensation, which means the error distribution is closer to the ideal zero. Moreover, the proposed method is also suitable for improving the accuracy of an INS and star tracker integrated navigation system.

## Figures and Tables

**Table 1:** Main performance specifications of the IHDST.

Parameter	Value
Accuracy (″)	<1 (pointing), <10 (rolling) (1*σ*)
Sensitivity (Mv)	9.0
Maximum angular rate (°/s)	25
FOV (°)	20 × 20
Update rate (Hz)	25
Power consumption (W)	5
Weight including baffle (kg)	1.4
Dimensions including baffle (mm)	130 × 130 × 285

FOV: field of view.

**Table 2:** Optimal estimations of the installation and misalignment errors.

Euler Angles of the Installation Error (*γ_B_*, *θ_B_*, *φ_B_*) (°)	Euler Angles of the Misalignment Error (*γ_R_*, *θ_R_*, *φ_R_*) (°)
(−0.0579, −0.4665, 0.7979)	(−0.6590, −0.0815, 0.4441)
